# Dimorphic enantiostyly and its function for pollination by carpenter bees in a pollen‐rewarding Caribbean bloodwort

**DOI:** 10.1002/ajb2.70148

**Published:** 2026-01-22

**Authors:** Steven D. Johnson, Jeremy J. Midgley, Luis G. Bocourt‐Hernandez, F. G. Loiret, Patricia Ortega‐Rodés, Nicola Illing

**Affiliations:** ^1^ Centre for Functional Biodiversity, University of KwaZulu‐Natal Pietermaritzburg 3209 South Africa; ^2^ Department of Biological Sciences University of Cape Town Cape Town 7701 South Africa; ^3^ Laboratorio Fisiología Vegetal, Depto. Biología Vegetal Facultad de Biología, Universidad de La Habana La Habana 10400 Cuba; ^4^ Department of Molecular and Cell Biology University of Cape Town Cape Town 7701 South Africa

**Keywords:** buzz pollination, flower colour, floral scent, floral signals, Haemodoraceae, heteranthery, mirror‐image flowers, pollinator, sexual conflict, Xylocopa

## Abstract

**Premise:**

Flowers that present their anthers and stigma in close proximity can achieve precise animal‐mediated pollen transfer, but risk self‐pollination. One evolutionary solution is reciprocal herkogamy. Reciprocity of anther and style positions among different plants (i.e., a genetic dimorphism) is common in distylous plants, but very rare in enantiostylous plants. We investigated the pollination and reproductive system of the enantiostylous Caribbean plant *Cubanicula xanthorrhizos* (Haemodoraceae).

**Methods:**

We assessed stylar orientation of flowers and conducted controlled pollination experiments. We used videography of flower visitors and pollen load analysis to determine the pollination mechanism. We also measured floral morphology, pollen production, spectral reflectance, and volatile emissions.

**Results:**

*Cubanicula xanthorrhizos* exhibits dimorphic enantiostyly with c. 50:50 left‐ to right‐styled morphs. Plants are self‐compatible, but pollinator dependent for seed production. Intra‐ and intermorph crosses are equally fertile. The nectarless flowers are pollinated by female carpenter bees (*Xylocopa cubaecola*) that collect pollen, often by sonication, from two centrally positioned yellow feeding anthers. An inconspicuous deflected pollinating anther deposits pollen on the side of the bee thorax, which contacts the stigma of the mirror‐image morph. A yellow‐orange “guide” on the white tepals appears to be a visual attractant. Flowers emit methoxy benzenoid volatiles that may also attract bees.

**Conclusions:**

Reciprocity of the style with a single pollinating stamen in *C. xanthorrhizos* appears to promote intermorph pollen export via “safe sites” on pollen‐collecting bees. This novel case of dimorphic enantiostyly contributes to understanding of the evolution of floral polymorphisms.

Efficiency of pollen export is an important component of male fitness in plants and can explain numerous floral modifications that have arisen in the radiation of angiosperms (Harder and Johnson, 2009; Johnson and Harder, 2023). Pollination efficiency is increased by traits that ensure that pollen is placed on specific parts of the pollinator body that are likely to contact stigmas. Precise pollen placement can be achieved by floral morphology such as zygomorphy that orientates the insect, resulting in repeatable pollinator positioning, and by tightly grouped anthers that increase precision of pollen placement (Armbruster, 2014).

While close proximity of the anthers and the stigma increases the precision of pollination, it can greatly increase the risk of self‐pollination within and among flowers (Barrett, 2002). Consequently, many plants separate the timing of floral organs (dichogamy) or their physical distance (herkogamy) (Lloyd and Webb, 1986; Webb and Lloyd, 1986; Barrett, 2002). However, herkogamy comes at the cost of reducing the precision of pollen transfer (Armbruster, 2014). A novel solution that has evolved in numerous plants is reciprocal herkogamy whereby the positions of anthers and the stigma alternate among flowers (Barrett, 1992; Simón‐Porcar et al., 2024). The most common form is distyly, which involves reciprocal changes in the heights of anthers and the style and is always a dimorphism with genetic differences among individual plants being either long‐ or short styled (Barrett, 1992). A less common form is enantiostyly, which involves lateral deflection of the style and, in many cases, also reciprocal deflection of one or more anthers (Jesson and Barrett, 2003; Barrett and Fairnie, 2024). Enantiostyly is usually a developmental polymorphism with both left‐ and right‐styled flowers on the same plant (monomorphic enantiostyly), but it can also take the form of a dimorphism with genetic differences among individuals (dimorphic enantiostyly) characterized by either left‐ or right‐styled plants (Jesson and Barrett, 2003; Barrett and Fairnie, 2024). Dimorphic enantiostyly is rare and has been recorded in only six plant species: *Heteranthera missouriensis* (formerly included in *H. multiflora*; Pontederiaceae; Jesson and Barrett, 2002a; Barrett and Fairnie, 2024) and four species of *Wachendorfia* (Ornduff and Dulberger, 1978; Jesson and Barrett, 2002b) and one in their sister genus, *Barberetta aurea* (Haemodoraceae) (Johnson et al., 2023).

Traits that reduce pollen losses during transport are critical for pollination efficiency (Johnson and Harder, 2023). Pollinator grooming, such as when bees move pollen to their scopae while in flight (Harder, 1990), can have highly deleterious effects on male fitness because pollen is moved away from body parts that are likely to contact stigmas (Thomson, 1986; Wolfe and Barrett, 1987). The problem of grooming can be particularly acute for plants that offer pollen as their sole reward because bees usually directly transfer the pollen that is collected as food to their scopae (Buchmann, 1983; Harder and Thomson, 1989; Parker et al., 2015).

Plants have mitigated the problem of pollen grooming by bees and other insects in several ways. Many plants place pollen on “safe sites” on insect that are not usually groomed or are difficult to reach (Koch et al., 2017; Tong and Huang, 2018). Nevertheless, when female bees focus solely on pollen collection, it can be difficult for plants to avoid losing a substantial fraction of their pollen to grooming. One solution adopted by many plants that are pollen rewarding is a division of labor between anthers that supply pollen as food and those that place pollen on relatively safe sites on the body for purposes of siring (Vallejo‐Marín et al., 2009). Heteranthery is particularly well documented in plants with specialized feeding anthers that are vibrated by bees to release pollen (Luo et al., 2008; Vallejo‐Marín et al., 2009; Barrett, 2021). Feeding anthers are often centrally placed and conspicuous, while the pollinating anthers are often deflected and inconspicuous (Ushimaru et al., 2007; Barrett, 2021). Heteranthery is also often associated with enantiostyly in pollen‐rewarding flowers (Dulberger, 1981; Barrett and Fairnie, 2024; Johnson et al., 2025).

In this study, we focused on the Caribbean plant species *Cubanicula xanthorrhizos* (Haemodoraceae), which is narrowly endemic to Cuba (Pellegrini et al., 2020). Our interest in this species was sparked by a recent taxonomic revision with illustrations that indicated that the flowers have pronounced enantiostyly with a deflexed style, two centrally positioned lateral stamens and a median stamen that is deflexed opposite to the style (Pellegrini et al., 2020).

Recent studies have revealed that enantiostyly is not only a type of floral architecture that can reduce geitonogamy (Jesson and Barrett, 2002a; Johnson et al., 2023), but it can also represent a fine‐tuned mechanism for pollen transfer on the lateral body parts of insects, such as their wings (Minnaar and Anderson, 2021; Johnson et al., 2023, 2025). It is therefore critical to document the pollinators of enantiostylous plants to understand the function of the floral morphology and display traits. On the basis of its floral traits, including the apparent division of the androecium into feeding and pollinating anthers, we hypothesized that *C. xanthorrhizos* is pollinated by large female bees.

The aims of this study were (1) to investigate whether enantiostyly in *C. xanthorrhizos* is dimorphic (i.e., a genetic polymorphism), (2) to quantify the reciprocal positioning of floral organs and to assess heteranthery, including the dimensions and pollen production of lateral and median stamens, (3) to determine whether the species is self‐compatible and is reliant on pollinators for seed production, (4) to identify effective pollinators and determine the mechanisms of pollen transfer among flowers, and (5) to quantify floral display traits including spectral reflectance and scent chemistry.

## MATERIALS AND METHODS

### Study species

**Figure 1:**
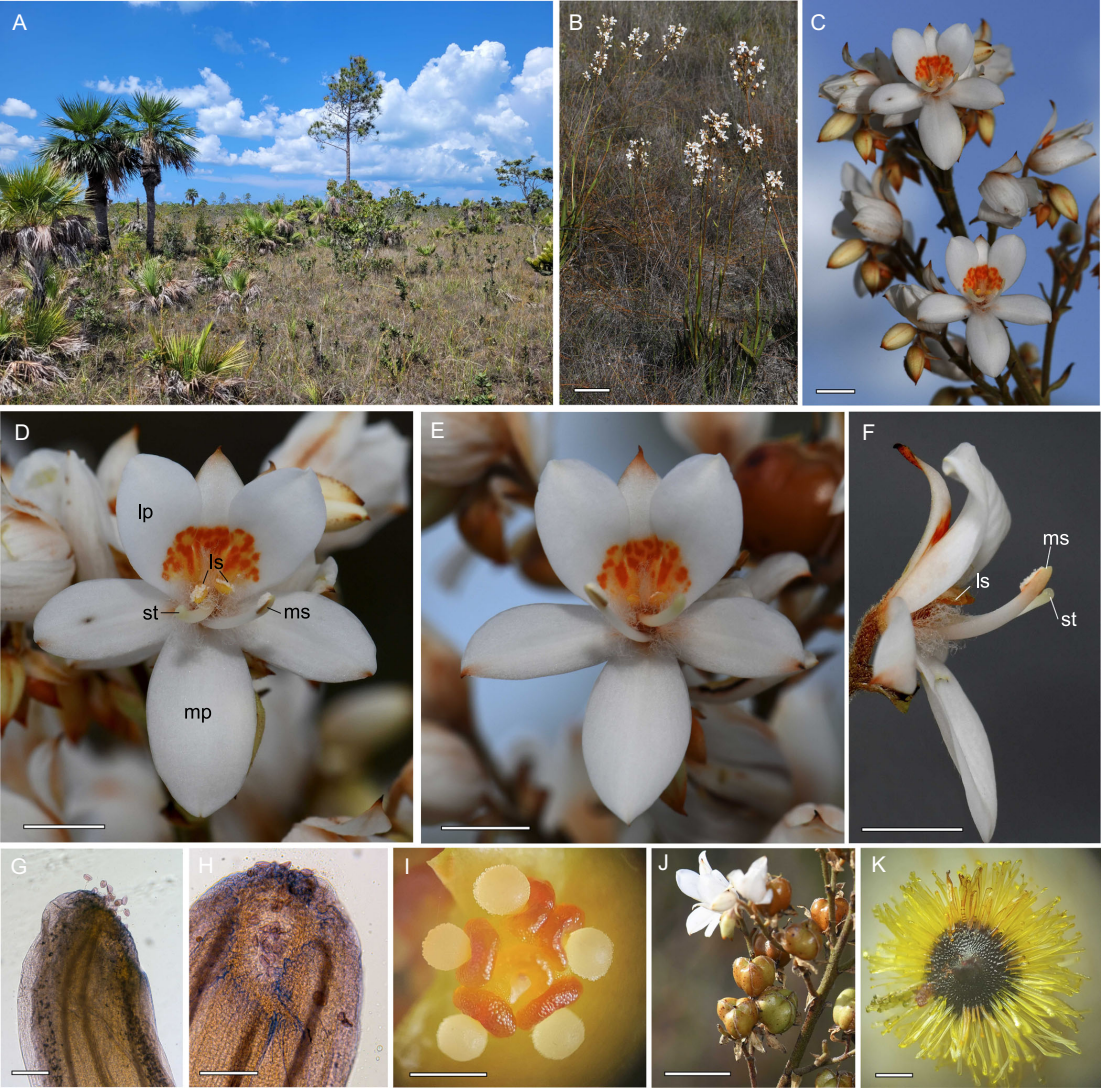
Habitat and floral morphology of *Cubanicula xanthorrhizos*. (A) Habitat in open pine woodland on the Isle of Youth, Cuba. (B) Inflorescences and leaves of flowering plants. (C) Inflorescence with two fully open flowers and older wilted flowers with a persistent perianth. (D) Left‐styled flower. (E) Right‐styled flower. (F) Side view of right‐styled flower showing the alignment of the longer median pollinating anther and stigma and the very short length of the centrally‐positioned lateral stamens which are enclosed in a mat of hairs. (G) Style showing the inward facing position of the stigma. (H) Crateriform stigma with several pollen grains of *C. xanthorrhizos*. (I) Dissected ovary showing unusual placentation of the ovules, five of which are developing into seeds. (J) Fruits. (K) Seed showing the distinctive fringe of hairs. Abbreviations: lp = lateral petal, ls = lateral stamens, mp = median petal, ms = median stamen, st = style. Scale bars: B = 10 cm; C–F = 5 mm; G, H = 200 µm, I = 1 mm; J = 1 cm.

*Cubanicula xanthorrhizos* (C. Wright ex Griseb.) Hopper et al. (Haemodoraceae) was formerly placed in *Xiphidium*, but differs from that genus in several key traits, including the structure of the anthers (lateral dehiscence in *Cubanicula* vs. poricidal in *Xiphidium*) and the seeds (large and coarsely hairy on the margins in *Cubanicula* vs. small and tuberculate in *Xiphidium*) (Pellegrini et al., 2020). *Cubanicula* is a monotypic genus that is in the same clade as *Xiphidium* and *Pyrrorhiza* (also monotypic), which is distinct from the clade of *Schiekia*, *Wachendorfia*, and *Barberetta* (Hopper et al., 2009; Pellegrini et al., 2020). The phylogenetic relationships among *Cubanicula*, *Xiphidium*, and *Pyrrorhiza* are currently not resolved; however, *Cubanicula* and *Pyrrorhiza* share a number of distinctive traits, including unusual seeds with a hairy fringe (Pellegrini et al., 2020). *Cubanicula xanthorrhizos* is known only from open pine woodland in the Province of Pinar del Río Cuba on the far west of the main island of Cuba and on the Isla de la Juventud (Island of Youth) (Figure 1A), which is situated approximately 60 km south of the western tip of the main island. Pellegrini et al. (2020) proposed that the species be classified as Endangered according to criteria of the Internation Union for Conservation of Nature.

Plants of *C. xanthorrhizos* produce c. 1–10 inflorescences that are typically about 1 m tall and are a complex, many‐branched thyrse (a racemose main axis with indeterminate growth and cymose secondary axes with determinate growth) that can bear up to 150 zygomorphic flowers, which are white with a conspicuous orange patch across the three upper tepals (Figure 1B–E). Simpson (1993) noted that *Cubanicula xanthorrhizos* (as *Xiphidium xanthorrizon*) lacked septal nectaries. The flowers have two lateral stamens with yellow anthers that are centrally located, and a longer deflected median stamen with a white anther (Figure 1D–F).

### Study site

We studied a small population of c. 45 plants, of which c. 15 had inflorescences, near Sandino (latitude, longitude: 22.11049, –84.16269) in the Pinar del Rio Province in 2024, and a large population (>1000 flowering plants) near Siguanea (21.64183, –82.97556) on the Isla de la Juventud, Cuba in 2024 and 2025. Plants grow on sandy soil in among scattered pines (*Pinus caribaea* Morelet) and bottle palms (*Colpothrinax wrightii* Schaedtler) (Figure 1A). Other flowering plants in the same community on the island include *Miconia delicatula* A. Rich. (Melastomataceae) and *Byrsonima wrightiana* Nied. (Malphigiaceae). Experiments and observations were carried out during peak flowering over 3 days in 2024 (30–31 May, 3 June 2024) and 5 days in 2025 (6–11 June 2025). A voucher specimen 93936 of *C. xanthorrhizos* was deposited at Herbario del Jardín Botánico Nacional Johannes Bisse, Cuba.

### Flower dimensions and enantiostyly

We assessed daily floral display (the number of simultaneously open flowers) by recording all open flowers on 44 inflorescences, each on a separate plant, and also counted the number of inflorescences on 20 plants. To determine flower longevity and the time intervals between anthesis of successive flowers on each inflorescence branch, we marked 20 buds on 20 cut inflorescences kept with their stems in water and also 41 buds on 15 intact bagged plants in the field and examined them daily to record the timing of anthesis and wilting and the anthesis of the next flower on each inflorescence branch.

To assess floral dimensions in the Isla de la Juventud population, we selected 25 flowers, each on a separate plant, and measured flower width and the vertical distance from the top of the lowermost tepal to the tip of the uppermost tepal, the length and width of the upper and lower tepals, the length of the style and each stamen, the length of the anthers, and the distance between the stigma and the median stamen. The morph (left‐ or right‐styled) was also recorded for each plant. We obtained grand means for each trait and also used Gaussian generalized linear models (GLMs) with an identity link function to determine whether floral dimensions varied among morphs. Significance was assessed using likelihood ratios. Unless otherwise stated, statistical tests were implemented in SPSS 29 (IBM, Armonk, NY, USA).

To establish whether *C. xanthorrhizos* is enantiostylous and whether style and median stamen orientation varies among flowers on inflorescences, we recorded style and stamen orientations of 47 flowers on five plants at the Pinar del Rio and 390 flowers on 98 plants at the Isla de la Juventud site.

The Isla de la Juventud site has a population of more than 1000 plants. The frequencies of left‐ and right‐styled plants were counted for 75 plants in 2024 and 68 plants in 2025 at this site. To test whether morph ratios of plants in this population deviated significantly from 50:50 we used binomial tests.

To assess the degree of reciprocity of the floral organs, we photographed 25 flowers, each taken from a separate plant, from the front and the side with a steel ruler for scale. These photographs allowed us to calculate three‐dimensional *x*, *y*, and *z* coordinates of the floral organs relative to the center of the flowers, as shown diagrammatically by Simón‐Porcar et al. (2024) and Johnson et al. (2025). We obtained these measurements using ImageJ (Schneider et al., 2012) calibrated according to the ruler included in each photograph. The coordinates were used to calculate a three‐dimensional standardized total inaccuracy metric, based on the inaccuracy metric proposed by Armbruster et al (2017). Calculations were performed using the R package FlowerMate (Simón‐Porcar et al., 2024). The metric 3DM^2^STI, which is the three‐dimensional version of Armbruster et al.'s (2017) inaccuracy metric, represents the overall departure from optimal positioning and the imprecision (within population variance), with each of these measures being equally weighted. Flowers with perfect matching of reciprocal organs without any variance among individuals would thus have zero inaccuracy.

### Pollen and ovule production

To assess pollen production in the deflected median anther versus the centrally located lateral anthers, we covered 20 inflorescences in fine mesh bags and after the flowers had reached anthesis, we removed dehisced anthers from 20 flowers, each from a different plant, with forceps and placed them in 0.5‐mL microfuge tubes. The anthers from the lateral stamens and the median stamen were placed in different tubes. We then added 400 µL of 70% v/v ethanol and 2 µl of 1% v/v aqueous methylene blue to each microfuge tube. After sonicating each microfuge tube for 30 min in a water bath, we agitated it for 20 s using a mechanical shaker and counted all the pollen grains in 10‐µL subsamples using a compound microscope. The total amount of pollen per anther was determined from the ratio of the subsample to the original volume. A second subsample was taken from 13 microfuge tubes to assess the replicability of the counting method. This analysis showed a high statistical correlation between subsamples (Pearson *r* = 0.81, *P* < 0.001), and a single subsample was thus deemed sufficient. We measured the size of 1–5 pollen grains in each of the pollen samples (in total 37 pollen grains from the short lateral stamens and 26 from the longer median stamens) using a compound microscope at 400× magnification and the measuring tools in Zeiss Zen software (Zeiss, Oberkochen, Germany).

We compared pollen production among stamen types using generalized estimating equations (GEEs) incorporating a negative binomial distribution and log link function. Significance was assessed with score statistics. Flower identity was treated as the subject with an exchangeable correlation matrix to account for lack of independence among stamens from the same flowers, and stamen type was treated as a fixed effect. For comparison of pollen size among stamen types, we altered the GEE to incorporate a Gaussian distribution and identity link function.

An additional 13 flowers, each from a different plant, were dissected to assess the number of ovules available per flower. We also mounted 23 stigmas of naturally pollinated flowers in fuchsin gel to determine the surface structure of the stigma and typical natural pollen loads on stigmas.

### Controlled pollination experiments

To assess the degree of self‐compatibility and pollinator dependence of *C. xanthorrhizos*, we conducted controlled pollinations using 529 flowers on 53 plants that were enclosed from the flower budding stage in fine mesh bags that excluded insects. We assigned flowers on these plants to the following treatments: (1) unmanipulated as a test for autonomous self‐fertilization, (2) self‐pollination with pollen from the centrally positioned lateral stamens of a flower on the same plant to test for self‐compatibility, (3) intermorph cross pollination with pollen from the longer median stamen of a different plant to serve as a reference for other treatments, (4) intramorph cross pollination with pollen from the longer median stamen of a different plant to test for heteromorphic incompatibility, (5) intermorph cross pollination with pollen from the centrally positioned lateral stamens of a different plant to test whether the pollen from these stamens is viable. We recorded the proportion of flowers that set fruit and the number of seeds per fruit for each treatment group. We also recorded fruit set and seeds per fruit for 336 naturally pollinated flowers on an additional 20 plants that were not enclosed in mesh bags.

Although we combined different treatments on 35 plants, we were able to apply only the unmanipulated treatment to flowers on 18 of the plants and a further 20 plants had only the natural pollination treatment. Due to the clustering of treatments on plants and the highly unbalanced design, we used generalized estimating equations (GEEs) with plant as the subject with an exchangeable correlation matrix to adjust for statistical non‐independence among flowers on a plant and included treatment as the fixed effect. Stylar orientation of flowers was included as an additional fixed effect in an initial model, but was not significant and was dropped from the final model. Significance was assessed using score statistics. In the case of the proportion of flowers that set fruit, we used binomial models with a logit link function, and in the case of seed set per fruit and per flower, we used negative binomial models with a log link function. Post hoc comparisons of means was based on the sequential Šidák method (Kirk, 1995).

### Pollinator behavior and pollen loads

We observed pollinators over 4 days (7 to 10 June 2025), typically from 08:00 until 13:00 hours. Additional observations from 06:00 to 18:00 were also made while working in the population on 6 June and 6 July 2025 Observations were conducted by three observers on 6–8 June and two observers on 9–10 June and 6 July, for a total of 65 observer hours. We noted the identity of flower visitors and caught representative samples with a hand net. We monitored inflorescences from two plants using continuous video recorded with two Mobius maxis cameras (Huizhou tuopu xunshi Technology Co., Huizhou City, China) from 09:10 to 12:10 hours for 1 day. We noted the behavior of all flower visitors, including pollen collection, and recorded behavior of visitation sequences on 57 plants involving 18 insects using a Sony AX53 camcorder in HD format (1020 × 1080 pixels, 120 frames per second). Videos were then slowed by 50×, allowing us to measure the time taken for flower visitors to handle flowers and the contact between floral organs and their body. Bees sometimes “sonicated” the flowers. Sonication by bees involves the use of pulses of energy from the indirect wing muscles to assist in mechanical release of pollen (Buchmann, 1983). Sonication is usually associated with buzz‐pollination of flowers with poricidal anthers, but is also sometimes used by bees to collect pollen from flowers with non‐poricidal anthers (Buchmann, 1985). For videos with audible sonication by bees, we estimated the fundamental frequency (in Hz) and duration of the associated audio files (48 kHZ sampling rate) using the plot Fast Fourier Transform (FFT = 4.096 Hz, Hamming window) spectrum function in the publicly available software Audacity v. 3.7.5 (Audacity Team, 2025). The fundamental frequency for wingbeats and sonication was taken as the lowest frequency in the harmonic series associated with each behavior (De Luca and Vallejo‐Marín, 2013; Vallejo‐Marín, 2019). To test Simpson's assertion that the species lacks nectaries and produces pollen as the sole reward, we inserted fine microcapillaries into flowers that had been previous bagged for 24 h and used a dissecting microscope to check the microcapillaries for liquid consistent with the presence of nectar.

We measured the body and wing length of flower visitors and used small (c. 8 mm^2^) blocks of fuchsin gel to swab pollen from the side of the thorax between the front and middle legs because this site is contacted by anthers. We also separately swabbed the scopae of bees (all flower visitors were bees) and the vials in which they were stored to gather any pollen they had collected. We used reference slides of *C. xanthorrhizos*, *M. delicatula*, and *B. wrightiana* to identify pollen grains of these species and also assigned remaining pollen that could not be identified to morphological categories. We counted all *C. xanthorrhizos* pollen grains on slides of pollen from the site between the front and middle legs. To establish the proportion of *C. xanthorrhizos* pollen in the samples, we subsampled all pollen grains (range 210–1953, median 362) in transects across the slides and assigned each pollen grain to *C. xanthorrhizos* or *M. delicatula* or a different morphospecies (pollen of *B. wrightiana* was not found on the *Xylocopa* bees). We compared the proportion of *C. xanthorrhizos* pollen grains in the pollen loads on different sites on the bodies of flowers visitors using an events/trials binomial GLM that was corrected for overdispersion. Because only four bees were captured on *Cubanicula*, bee identity was included as a fixed effect rather than as a random effect.

### Spectral reflectance of flower parts

To assess visual signalling of flowers, we measure spectral reflectance (in the 300–700 nm wavelength range) of tepals, the orange guide patch on the tepals, the style, the centrally positioned lateral anthers, the anther of the longer median stamen, and the pollen from both types of anthers. We used a reflectance spectrometer (Ocean Optics S2000, Ocean Optics, Duiven, Netherlands) with a portable light source (Ocean Optics DT‐mini‐2GS) and a 200 µm reflectance probe (Ocean Optics QR 200‐7‐UV‐BX) suitable for measurements of reflectance from small objects. Measurements of the spectral reflectance of pollen were obtained by dabbing pollen onto a small patch of clear sellotape until it was uniformly covered. We calculated the loci of spectra in the color hexagon (Chittka, 1992), which is based on the excitation of honeybee color receptors and a widely accepted model of bee color vision that is supported by behavioral experiments with a range of bee species. Our calculations incorporated a general green leaf background spectrum and D65 daylight illumination spectrum (Chittka, 1996).

### Floral scent

To characterize the floral volatiles emitted by *C. xanthorrhizos*, we enclosed inflorescences in polyacetate bags (Kalle, Wiesbaden, Germany) and used PAS‐500 personal air samplers (Spectrex, Palo Alto, CA, USA) to pump air from the bags and through small glass cartridges containing 1.5 mg each of carbotrap B (20–40 mesh; Sigma‐Aldrich, Merck, Darmstadt, Germany) activated charcoal and Tenax TA (60/80; SupelcoTM, Merck, Darmstadt, Germany) at a realized flow rate of 50 mL/min. Samples were taken from eight intact inflorescences (i.e., attached to plants) and from a further three cut inflorescences. Sampling of cut inflorescences commenced immediately after they were removed from plants and took place in the shade (under palm trees) at the same time (10:00–11:00 hours) as the sampling from intact inflorescences. Sampling of cut inflorescences in the shade was done because of concerns that temperatures inside plastic bags on intact inflorescences may have been too high. We recorded the number of flowers on each sampled inflorescence and the sampling time (median 90 min) to calculate the rate of scent production on a per flower basis. Control samples were taken for the same duration from empty bags both in the sun and in the shade.

To analyze the headspace samples, we used coupled gas chromatography and mass spectrometry (GC‐MS) with a Varian CP‐3800 GC (Varian, Palo Alto, CA, USA) equipped with a 30 m long × 0.25 mm internal diameter and Scion‐WaxMS polar column (0.25 μm film thickness, Scion, Goes, Netherlands) coupled to a Bruker 300 quadrupole mass spectrometer (Bruker, Billerica, MA, USA). Samples were thermally desorbed using a ChromatoProbe thermal desorption device in a Varian 1079 PTV injector port. The flow rate for the helium carrier gas was 1 mL min^−1^. The injector was held at 40°C for 2 min with a 20:1 split and then increased to 200°C at 200°C min^−1^ in splitless mode for thermal desorption. After a 3‐min hold at 40°C, the temperature of the GC oven was increased to 240°C at 200°C min^−1^ and held at 240°C (with a 1:100 split) for the remainder of the run. To obtain additional data for compound identification, we also ran one of the samples from a cut inflorescence through a Bruker BR‐5 semi‐standard non‐polar column. Compounds were identified using Varian MS Workstation software (version 7.0) in conjunction with the NIST 2020 mass spectral library (NIST, Gaithersburg, MD, USA). Library identifications were based on mass spectra matching and comparisons of calculated linear (non‐isothermal) *n*‐alkane retention indices (Van den Dool and Kratz, 1963) with published values for both polar and non‐polar columns. In some cases, additional verification was obtained by injection of authentic standards. The amount of scent emitted by flowers was estimated from comparison of peak areas in the samples with the peak area resulting from injection of a known amount of methyl benzoate. Compounds present at similar abundance in control samples taken from the empty bags were considered to be contaminants and were excluded from the analyses.

## RESULTS

### Flower design, dimensions, and enantiostyly

All of the marked flowers of *C. xanthorrhizos* remained open for just 1 day. Anthesis in cut inflorescences with their stems in water took place at approximately 04:00 hours, and the perianth was fully open by 07:00. However, anther dehiscence occurred only at c. 09:00 and coincided with noticeable production of floral scent and the commencement of pollinator activity (see below). Flowers closed by the evening and remain closed the following morning, even on plants from which pollinators were excluded, indicating that 1‐day floral longevity is programmed and not a consequence of pollination. Anthesis of the next flower in the sequence on each inflorescence branch occurred c. 4 days after the previous flower has closed. The consequence of this staggered pattern is that the daily display size was just 3.56 ± 0.24 flowers of open flowers per inflorescence, despite the numerous branches on each inflorescence. The average number of inflorescences per plant was 4.85 ± 0.71, thus translating to a daily display of c. 17 flowers per plant. Closed flowers retained their perianth for several days contributing to the overall visual display of the inflorescences (Figure 1B,C). The closed tepals of pollinated flowers covered the developing fruit capsule and only dried out once the fruit capsule reaches maturity (Appendix S1). Flowers that were not fertilized, abscised from the plant within a few days.

The overall mean (±SE) width of *C. xanthorrhizos* flowers was 20.4 ± 0.61 mm, and the vertical distance from tip of the lowermost tepal to the tip of the uppermost tepal was 20.1 ± 0.41 mm. The upper tepals were 8.7 ± 0.22 mm long and 5.2 ± 0.12 mm wide. The middle lower tepals were slightly longer and narrower; 11.4 ± 0.29 mm long and 5.8 ± 0.14 mm wide. The orange‐yellow “guide” patch that spans the three upper tepals was 4.2 ± 0.18 mm tall and 4.9 ± 0.13 mm wie. The horizontal deflection from the flower center was 2.8 ± 0.09 mm for the style, 2.5 ± 0.09 mm for the median stamen and 0.85 ± 0.04 mm for the lateral stamens (Figure 1D, E; Table 1). The distance from the stigma to the anther of the deflected median stamen was 5.3 ± 0.16 mm. The length of the style was 8.0 ± 0.11 mm. There were significant differences between the lengths of the deflexed median stamen and the centrally located lateral stamens (8.7 ± 0.14 vs. 3.7 ± 0.10 mm, Table 1) and smaller, but significant differences between the lengths of the median anther and lateral anthers (1.7 ± 0.05 mm vs. 1.9 ± 0.03 mm, Table 1). For all of the morphological traits that were measured, there were no significant differences in dimensions between left‐ and right‐styled flowers (*P* > 0.1 in all cases).

**Table 1:** Characteristics of the centrally located lateral feeding stamens versus the deflected median pollinating stamens of *Cubanicula xanthorrhizos*. Values are means ± SE. Values in parentheses are the sample sizes in terms of number of flowers and plants, respectively. Pollen counts are per anther. See Figure 5 for differences in anther spectral properties.

Trait	Lateral stamens (*N*: flowers, plants)	Median stamen (*N*: flowers, plants)	*χ* ^2^	*P*
Horizontal deflection (mm)	0.86 ± 0.03 (25, 25)	2.5 ± 0.07 (25, 25)	4.77	0.03
Stamen length (mm)	3.7 ± 0.10 (20, 20)	8.7 ± 0.14 (20, 20)	21.4	<0.001
Anther length (mm)	1.9 ± 0.03 (20, 20)	1.7 ± 0.05 (20, 20)	8.7	0.003
Pollen grains	1499 ± 369 (20, 20)	3009 ± 818 (20, 20)	2.11	0.14
Pollen length (µm)	44.6 ± 0.82 (26, 20)	42.0 ± 1.11 (37, 20)	3.17	0.07
Pollen width (µm)	30.9 ± 0.98 (26, 20)	29.7 ± 0.95 (37, 20)	4.97	0.08

We found dimorphic enantiostyly in *C. xanthorrhizos*. The orientation of the style and median stamen was fixed (all flowers were either left‐ or right‐deflected) in all five plants examined at Pinar del Rio and the 98 plants examined at the Isla de la Juventud site. Too few flowering plants (*N* = 5, four left‐styled and one right styled) were found at Pinar del Rio to conduct statistical tests of morph ratios. Observed morph ratios at the Isla de la Juventud site did not differ from a 50:50 ratio and were 59% right styled (*N* = 75, *P* = 0.164) in 2024 and 50% right‐styled (*N* = 68 plants, *P* = 1.0) in 2025.

**Figure 2:**
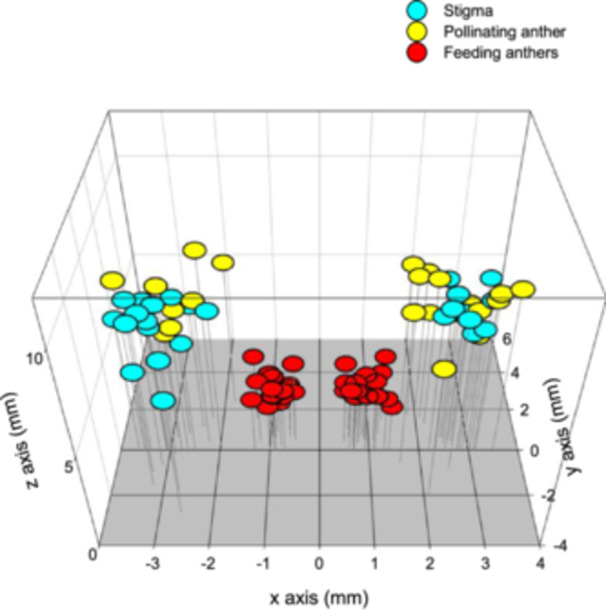
Coordinates of anthers and the stigma of left‐ and right‐styled *Cubanicula xanthorrhizos* flowers plotted in three‐dimensional space. Note the considerable overlap of the coordinates of the (median) pollinating anthers and styles and the lack of overlap between the (lateral) feeding anthers and the stigma. Insert: Left‐styled flower showing measurement axes with origins in the centre of the flower at the base of the tepals.

The orientation of sepals and petals for flowers in a *C. xanthorrhizos* inflorescence was similar to that of *Wachendorfia* species (Appendix S2). However, this flower orientation is inverted in *Dilatris ixioides* (Appendix S2). The central feeding stamen of *D. ixioides* is thus developmentally analogous to the deflected median stamen of *C. xanthorrhizos* and *W. paniculata*. The lateral pollinating stamens of *D. ixoides* are developmentally analogous (homologous) to the feeding stamens of *C. xanthorrhizos*. The positions of the stigma and the anther of the median stamen are reciprocal, with considerable overlap in three dimensional space (Figure 2). By contrast the positions of the centrally positioned anthers of the lateral stamens do not coincide with the stigma. The three‐dimensional standardized total inaccuracy metric (3DM^2^STI) was very low for the intermorph median anther–stigma positioning (0.26) and lateral anthers–stigma positioning (1.54 and 1.92). The values for 3DM^2^STI were also relatively high for the intramorph median anther–stigma positioning (0.88) and lateral anthers–stigma positioning (1.94 and 1.55). These values indicate that the highest probability of pollen transfer is between the median anther and the stigma of the opposite morphs, as expected from enantiomorphic mirror image flowers.

The centrally positioned lateral anthers face upward and are covered in a mat of hairs (Figure 1D–F). Both median and lateral anthers showed lateral dehiscence of their locules. The stigma is situated on one side of the style (Figure 1G), facing toward the median anther, which also faces inward toward the center of the flower. The style is approximately 800 µm in diameter, and the stigma is approximately 300 µm in diameter and has a crateriform structure surrounded by a circle of tubercules (Figure 1H). We recorded a mean of 3.4 ± 1.59 pollen grains (range = 0–23) on stigmas; the majority (82%) of the 23 stigmas examined had no pollen grains deposited.

### Pollen and ovule production

Mean values recorded for pollen production were higher for the median anther than for the lateral anthers, but the means did not differ significantly between the two types of anthers on account of the high variance among samples (Table 1). Total pollen production per flower, estimated from the mean values for each anther was c. 6007 grains. Pollen grains measured 43.3 ± 0.72 µm long and 30.3 ± 0.69 µm wide, and their size did not differ significantly among lateral and median anthers (Table 1).

The length of the superior ovary was 2.27 ± 1.1 mm. Ovaries contained a mean of 22.6 ± 0.84 ovules (typically 7 or 8 per chamber arranged in a crownlike pattern of placentation and swollen placental attachments (Figure 1I). The pollen to ovule (P:O) ratio was 266.

### Controlled pollination experiments

**Figure 3:**
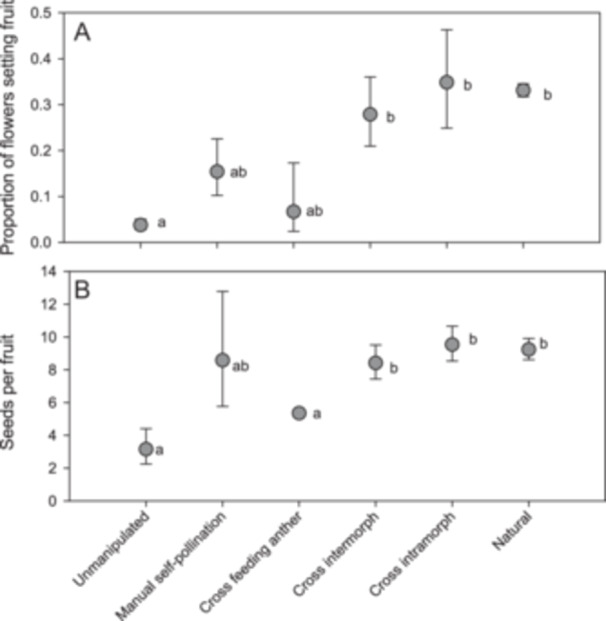
Results of controlled pollination experiments involving *Cubanicula xanthorrhizos*. (A) Proportion of flowers that set fruit. (B) Number of seeds produced per fruit. Flowers were hand‐pollinated with pollen from the (median) pollinating anther, unless otherwise stated. Means that share letters are not significantly different (Šidák test, *P* > 0.05).

Approximately 33% of flowers in the population naturally set fruit, which contained an average of 9.2 seeds (Figure 3). Very similar values of fruit and seed set were obtained for flowers that were manually cross‐pollinated using pollen from the median anther (Figure 3). Fruit set did not vary according to whether the pollen used for cross pollination came from plants of the same morph or different morphs, indicating a lack of heteromorphic compatibility (Figure 3). Although cross pollination with pollen from the centrally positioned lateral anthers resulted in just 6% of flowers setting fruit, this value was not differ significantly from values obtained for flowers cross‐pollinated with pollen from the median anther and those from which pollinators were excluded (Figure 3). However, the number of seeds per fruit in flowers cross‐pollinated with pollen from the lateral anthers was significantly reduced in comparison with flowers cross‐pollinated with pollen from the median anther, suggesting that fertility of pollen from the lateral anthers may be lower than that of pollen from the median anthers. Manual self‐pollination resulted in fruit set in 15% of flowers which did not differ significantly from that for cross‐pollinated flowers indicating that *C. xanthorrhizos* is genetically self‐compatible. Fruit set occurred in just 3.7% of flowers from which pollinators were excluded, and these fruits contained just three seeds on average. These values were significantly lower than those obtained for cross‐pollinated flowers and for naturally pollinated flowers (Figure 3), indicating that *C. xanthorrhizos* is reliant on pollinators for seed production. We noted that anthers did not contact the stigmas during flower closure (the median distance between anthers and the stigma in closed flowers was 1.9 mm, *N* = 17). Therefore, the few fruits that were produced in plants from which pollinators were excluded were probably due to self‐pollination from bagging fabric rubbing over the flowers in the wind. We counted 31.1 ± 2.36 fruits per inflorescence on naturally pollinated plants, and given that we also recorded 4.85 ± 0.71 inflorescences per plant, we estimate that a total of about 150 fruits were produced per plant, on average.

### Pollinator behavior and pollen loads

**Figure 4:**
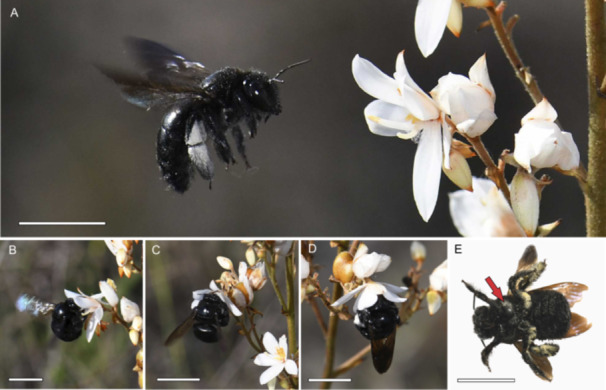
Visits to flowers of *Cubanicula xanthorrhizos* by pollen‐collecting females of the carpenter bee *Xylocopa cubaecola*. (A) Approach to a flower. (B) Hovering while grasping the flower. (C) Front legs raised over the central anthers and the lateral anther and stigma contacting the bee on either side of the thorax between the front and middle legs. (D) Hanging below the flower with wings closed momentarily and front legs scraping pollen out of the central anthers. (E) Site of pollen deposition by the lateral anther. Pollen deposited on this site by the lateral anther of a plant of one morph will be picked up by the style of plants of the opposite (mirror image) morph. The polystyrene background behind the bee has been digitally removed for clarity. Scale bars = 10 mm.

Flowers of *C. xanthorrhizos* were visited almost exclusively by the carpenter bee *Xylocopa cubaecola* (Figure 4A). We observed foraging bouts by 55 *X. cubaecola* bees, involving visits to several hundred flowers. Almost all bee visits to the flowers took place between 09:00 and 12:00 hours. These bees visited a mean (±SE) of 1.55 ± 0.10 flowers per inflorescence (*N* = 56), and the duration of each flower visit that could be timed was 440 ± 22.7 ms (*N* = 54). The bees grasped the flower with their middle and back legs and collected pollen from the centrally positioned lateral anthers (Figure 4B–D; Appendix S3), which are hereafter referred to as the feeding anthers. They did not collect pollen from the median anther (hereafter, the pollinating anther). Bees often sonicated the pollen when settled on the flowers with their wings closed. The mean (±SE) duration of sonication per flower was 290 ± 8.0 ms. The fundamental frequency of the bee wingbeat was c. 143 Hz, and the fundamental frequency of the sonication was c. 300 Hz, but was difficult to measure with accuracy because the volume of the sonication was low. However, sonication resulted in a very clear harmonic series that appeared in all of the audiofiles and corresponded to the audible sound of sonication when bees had folded their wings after grasping the flower (Appendix S4).

We did not observe any attempts by bees to probe for nectar, and microscopic examination of microcapillaries inserted into flowers did not provide any evidence of liquid consistent with nectar. The flowers thus appear to offer pollen as the sole floral reward.

While bees collected pollen from the feeding anthers, the pollinating anther and the stigma contacted the side of the bee's thorax between its front and middle legs (Figure 4B; Appendix S3). Visits to flowers of both morphs resulted in two distinct patches of pollen on these sites, anatomically representing the mesepisternum section of the mesopleuron of the thorax (Figure 4E). We captured four individuals of *X. cubaecola* on flowers of *C. xanthorrhizos*, and pollen swabs taken from the mesepisternum contained a mean (±SE) of 3588 ± 1409 *C. xanthorrhizos* pollen grains. *Cubanicula* pollen grains made up 98.4 ± 0.6% of the pollen on the mesepisternum versus 78.6 ± 3.7% of pollen on the rest of the body (*χ* ^2^ = 10.8.1, *P* < 0.001). The second most abundant pollen was of *M. delicatula*, making up 4.4 ± 1.6% of the pollen on the mesepisternum versus 26.6 ± 5.6% of pollen on the rest of the body of bees captured on *Cubanicula* (*χ* ^2^ = 58.6, *P* < 0.001). In the case of a single individual of *X. cubaecola* captured while it was sonicating flowers of *M. delicatula* about 800 m from the study population, pollen of *M. delicatula* made up 99.3% of the pollen on the body.

The only other insects observed on the flowers of *C. xanthorrhizos* were a single individual of *Centris poecila* (Appendix S5) and several small halictid bees (*Lasioglossum parvum* and *L. gundlachii*) that collected pollen without contacting the stigma. *Centris* bees were also observed collecting oil from flowers of *Byrsonima wrightiana* (Malphigiaceae) (Appendix S5) and sonicating flowers of *Miconia delicatula* (Melastomataceae) (Appendix S5).

### Spectral reflectance of flower parts

**Figure 5:**
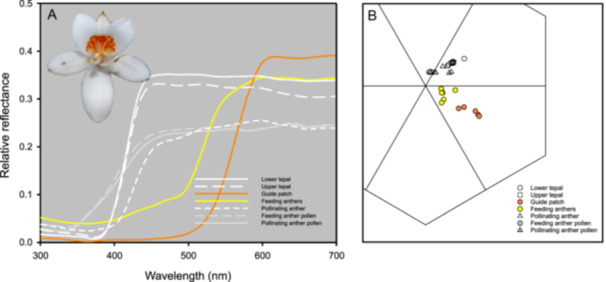
Visual signals of *Cubanicula xanthorrhizos*. (A) Relative spectral reflectance (as proportions) for flowers of *Cubanicula xanthorrhizos*. Curves are mean values for flower parts. (B) Loci of spectra for various flower parts in the color hexagon, which is a representation of colors as they may be discriminated by the visual system of bees. The background color of leaves falls in the center of the hexagon.

The upper and lower tepals of *C. xanthorrhizos* are white and lack UV reflection (Figure 5A). The guide patch across the three upper tepals consists of light orange flecks over a yellow background and is also UV‐absorbing (Figure 5A). The feeding anthers are light‐yellow (Figure 5). The pollinating anther is white with a reflectance pattern similar to that of the tepals (Figure 5A). The pollen of the pollinating and yellow feeding anthers is light‐grey with some reflection in the UV‐wavelengths (Figure 5A). The loci of the pollen spectra were close to the center of the color hexagon (Figure 5B), indicating that pollen would not be conspicuous to bees. The loci of the UV‐absorbing white tepals were farther from the center of the color hexagon and also quite distinct from the loci of the guide patches and the yellow feeding anthers (Figure 5B).

### Floral scent

Flowers of *C. xanthorrhizos* had a scent that is very weak and only discernible to humans (at a distance of c. 10 cm from the flower) from about 09:00 until 15:00 hours. The mean (±SE) emission rate of scent based on 10 samples taken in the late morning was 9.4 ± 3.0 ng per flower per hour. The scent consisted of a blend of seven structurally similar benzenoid compounds (Table 2; Appendix S6), including two with methoxy groups, and was dominated by benzyl acetate (36.9%) and 1‐ethenyl‐4‐methoxybenzene (31.3%). The scent blend was consistent (i.e., repeatable) in all of the samples that were analyzed. Compounds in the scent samples from inflorescences attached to plants (*N* = 8) and from cut inflorescences (*N* = 2) that were run on a polar column did not differ significantly (ANOSIM, *P* = 0.15), and the samples were thus combined for analysis. Our compound identifications based initially on library matching of mass spectra were further supported by the matching of Kovats retention indices with previously published values for both polar and nonpolar columns (Table 2).

**Table 2:** Chemical composition of floral scent of *Cubanicula xanthorrhizos*. LRI = linear retention index (Van Den Dool and Kratz, 1963), as calculated for 10 samples run using a polar column and one sample run using a nonpolar (DB5) column. LRI values in parentheses are mean values reported in the NIST 2020 database. Occurrence is the number of samples in which a compound was recorded in analyses using a polar column. Compounds with an asterisk (*) were confirmed by injection of authentic standards.

Compound	Polar LRI (NIST)	DB5 LRI (NIST)	Percentage of total peak area: Mean ± SE (Occurrence)
1‐Methyl‐4‐methoxybenzene	1417 (1432)	1020 (1021)	11.56 ± 1.75 (10)
1‐Ethenyl‐4‐methoxybenzene	1650 (1680)	1149 (1156)	31.28 ± 2.93 (10)
Benzyl acetate*	1701 (1697)	1159 (1164)	36.94 ± 2.7 (10)
2‐Phenylethyl acetate*	1790 (1803)	1252 (1258)	1.37 ± 0.21 (10)
Benzyl alcohol*	1842 (1865)	1028 (1030)	17.29 ± 1.72 (10)
2‐Phenylethanol*	1882 (1907)	1107 (1115)	1.1 ± 0.22 (10)
Ethyl 4‐ethoxybenzoate	2137 (NA)	1525 (1522)	0.45 ± 0.12 (9)

## DISCUSSION

### Enantiostyly and stamen deployment strategies

We identified a new case of dimorphic enantiostyly in flowering plants and elucidated how floral traits of *Cubanicula xanthorrhizos* are coordinated for precise pollen transfer on a small “safe site” on the bodies of pollen‐collecting bees. Our results provide a detailed example of the function of complex suites of floral traits for both attraction of pollinators and precise transfer of pollen among plants.

Dimorphic enantiostyly in *C. xanthorrhizos* is notable for several reasons. *Cubanicula* represents an extreme case of mirror‐image floral design in the Haemodoraceae with a deflected style opposed by a single pollinating anther. Among related taxa, similar floral designs with reduced lateral stamens and a single long median stamen are found in *Schiekia* in the Haemodoraceae (Pellegrini et al., 2020) and in *Heteranthera missouriensis* in the Pontederiaceae (Jesson and Barrett, 2002a), but their pollination biology is unknown. Other members of the Haemodoraceae with dimorphic enantiostyly, namely *Wachendorfia* and *Barberetta*, have three pollinating anthers, and at least one of these anthers is typically located on the same side of the flower as the style (Appendix S2). The term enantiostyly, which refers to mirror‐image style orientation, does not adequately describe the diverse stamen deployment strategies which often accompany enantiostyly. Ornduff and Dulberger (1978) used the term enantiomorphy which is gender‐neutral and less binary than enantiostyly. It is particularly suitable for Haemodoraceae, as the evolution of mirror‐image flowers in that family did not just involve changes in style orientation, but also a very complex range of stamen deployment strategies. Factors that influence these stamen deployment strategies include trade‐offs between avoidance for geitonogamy and the benefits of intramorph outcrossing (Jesson and Barrett, 2002a; Johnson et al., 2023), as well as pollen placement opportunities and exploitation of safe sites on the bodies of pollinators (Minnaar and Anderson, 2021; Johnson et al., 2023, 2025). Among enantiostylous genera in the Haemodoraceae, *Cubanicula* represents a highly developed enantiomorphic state involving reciprocity of the style and a single pollinating anther, while *Dilatris* is the least enantiomorphic with only the style showing left‐ versus right‐deflection (Johnson et al., 2025).

In *Wachendorfia* and *Barberetta*, the lateral stamens are of a similar length to the median stamen and serve a pollinating function (Minnaar and Anderson, 2021; Johnson et al., 2023). In *Cubanicula*, the lateral stamens are significantly shorter, and the anthers are presented centrally to provide pollen as a reward (i.e., feeding anthers). The median stamen is much longer and is presented on the opposite side of the flower from the deflected style and functions for pollination. *Dilatris* is also heterantherous, but with the important difference that the roles of the stamens are reversed relative to those in *Cubanicula*, with lateral stamens serving for pollination and the median stamen for feeding (Johnson et al., 2025).

The single pollinating anther of the median stamen of *C. xanthorrhizos* allows for pollen transfer that is potentially very precise. This precision was evident in the limited three‐dimensional inaccuracy for the intermorph lateral anther–stigma contrast, as well as the deposition of pollen on the side of the thorax between the front and middle legs of the bee visitors. This site (the mesepisternum) is notable for allowing for physical separation of pollen from left‐ and right‐style flowers and was characterized by very high pollen purity. (Pollen of *C. xanthorrhizos* comprised more than 98% of the pollen on this site on the body of bees.).

### Pollen collection by bees and floral signals

The carpenter bee *Xylocopa cubaecola*, which is endemic to Cuba and the Bahamas and is the only *Xylocopa* species in Cuba (Genaro, 2008), made up c. 95% of the recorded individual visitors to flowers of *C. xanthorrhizos* and 98.2% of the visitors capable of pollinating the flowers. The body of this bee fit *C. xanthorrhizos* flowers precisely, suggesting floral adaption for pollination by this particular bee species. Armbruster (2017) referred to cases where flowers have few insect visitor species on islands as “imposed specialization” because it may be the result of depauperate insect fauna, rather than floral filtering mechanisms We observed one visit to *C. xanthorrhizos* flowers by a *Centris* bee, and both *Xylocopa* and *Centris* bees buzz‐pollinated flowers of *M. delicatula*, which was common at the study site. Pollen loads on the *Xylocopa* bees comprised mainly pollen of *C. xanthorrhizos* and *M. delicatula*, confirming an ecological linkage between these two plant species, which were both regularly visited by these bees. Additional observations in multiple plant populations and across different years are needed to determine whether *Centris* bees play a significant role in the pollination of *C. xanthorrhizos*.

Heteranthery in *C. xanthorrhizos* involves the divergent length of the pollinating and feeding stamens, as well as the color and orientation of the anthers. Unlike heteranthery in *Dilatris*, which is associated with much more pollen production by the feeding anther compared to the lateral pollinating anthers, pollen production per anther in *C. xanthorrhizos* did not differ significantly between the median pollinating anther and the lateral feeding anthers. This similarity raises an interesting question about why bees did not gather pollen from the pollinating anther. One possibility is that the bright yellow of the feeding anthers and their backdrop of a large yellow‐orange guide pattern on the upper tepals are more visible to bees. By contrast, the pollen of the pollinating anther is white with some UV reflectance, which is a spectral pattern that renders objects similar to the background in the visual system of bees (Kevan et al., 1996). It is probably also harder for bees to gather pollen from anthers on long filaments than it is for them to gather pollen from anthers on short filaments.

An interesting feature of *C. xanthorrhizos* flowers is the spotted yellow‐orange guide, which spans the three upper tepals. Bees directed their antennae toward the guide pattern when approaching flowers (Appendix S3). The guide pattern may elicit sensory bias of female bees toward visual signals that are typical of anthers and pollen (Lunau, 1991; Lunau et al., 2024). It may also exploit behavior of female bees that is conditioned by grouped stamens of *M. delicatula* (Appendix S3), which were an important pollen source for *Xylocopa* and *Centris* bees at the study site. Buchmann (1985, p. 522) considered sonication of flowers with non‐poricidal anthers to be “probably triggered by morphological and visual cues similar to those found in buzz‐pollinated flowers”.

Bees collected pollen from the feeding anthers with their front legs and also regularly sonicated the flowers (Appendix S3). It is possible that sonication behavior was due to the similarity of floral signals between *C. xanthorrhizos* and *M. delicatula*, which has poricidal anthers. However, we think it more likely that sonication was used to extract pollen more efficiently from the feeding anthers of *C. xanthorrhizos*, which are covered with a dense mat of hairs that may make it difficult for bees to scrape pollen from the anthers with their front legs. The function of the mat of hairs is a matter of conjecture at this stage, and among the various possibilities are that the hairs around the anthers create the tactile impression of large amounts of pollen or that the hairs may serve as system for gradual dispensing of pollen to different flower visitors, such that all of the pollen is not simply collected by the first bee that visits the flowers.

The floral scent of *C. xanthorrhizos* comprised benzenoid compounds, including several methoxy benzenoids. Some methoxy benzenoids play a role in bee attraction (Dötterl et al., 2005), but the functions of the compounds we isolated in scent of *C. xanthorrhizos* are unknown. Surprisingly, given that bees are ubiquitous pollinators, the role of floral scent for attraction of bees to food‐rewarding flowers has been identified for only a few bee‐pollination systems (Dötterl et al., 2005; Milet‐Pinheiro et al., 2013; Schäffler et al., 2015; Kiepiel and Johnson, 2024). A general survey of flowers pollinated by carpenter bees revealed only weak chemical signatures that characterize flowers pollinated by these bees (Rabeschini et al., 2021). Some of the common benzenoids in the scent of *C. xanthorrhizos*, such as 2‐phenylethyl acetate and 2‐phenylethanol, elicit responses in bee antennae (Bisrat and Jung, 2022), but behavioral assays are mostly lacking. A general role of floral scent in pollen‐rewarding flowers is indicated by experiments that show that bees can associate floral scent with pollen rewards (Dobson et al., 1999; Arenas and Farina, 2012).

Floral scent chemistry is unknown for most other members of the Haemodoraceae, making it difficult to establish whether the scent of *C. xanthorrhizos* is unusual within the family. In *Xiphidium caeruleum*, which is closely related to *C. xanthorrhizos* but differs in having poricidal anthers, Buchmann (1980) found that floral scent was detectable only when flowers were placed in a glass jar to concentrate the scent, which appeared to emanate mainly from the apices of the tepals. The flowers of *X. caeruleum* were sonicated for pollen by female euglossine bees, but also visited by male euglossine bees, which appeared to collect scent from the flowers. Analysis of the chemical composition of the scent of *X. caeruleum* would therefore be of great interest for purposes of comparison with *C. xanthorrhizos*, which did not appear to attract any male bees.

One‐day flowers are found in many other Haemodoraceae such as *Wachendorfia*, *Dilatris*, and *Xiphidium* and in related families in the Commelinales such as Commelinaceae (Hardy et al., 2009). In *Wachendorfia*, the perianth is retained around the ovary, but wilts completely and has a jelly‐like consistency at the end of the first day of anthesis (Appendix S2). However, in *C. xanthorrhizos*, the tepals are retained in a turgid state for several days after the flowers have closed and are retained even after fruits are well‐developed (Appendix S1). In *Dilatris*, the tepals are retained for several weeks even though the flowers are sexually functional for just a single day. In both cases, the retention of the perianth likely contributes to the overall floral display of the plants. The functional significance of 1‐day flowers is not well understood (Primack, 1985; Stratton, 1989), but in the case of pollen‐rewarding flowers, may be related to their loss of attractiveness to pollinators when pollen is removed from feeding anthers in the first few hours after anthesis.

### Pollinator dependence

Our controlled pollination experiments showed that *C. xanthorrhizos* is highly dependent on pollinator visits for seed production. The experiments also showed that the species is self‐compatible and that crosses within and among morphs are equally fertile, as shown in several other enantiostylous members of the Haemodoraceae (Ornduff and Dulberger, 1978; Jesson and Barrett, 2002b; Johnson et al., 2023, 2025). Given that isoplethy of morphs in *C. xanthorrhizos* cannot be explained by heteromorphic incompatibility, morph ratios likely are maintained by negative frequency‐dependent selection due to the plant architecture that promotes intermorph pollination.

Our experiment included a test of the fertility of pollen from the feeding anthers, but the results were ambiguous, with only the number of seeds per fruit showing evidence of reduced fertility of pollen from the feeding anther (Figure 3B). Although the intensity of staining of pollen with methylene blue did not differ for pollen from the feeding and pollinating anthers, methylene blue stains only cytoplasmic cell contents and not nuclear material. Pollen from the feeding anthers was also identical in size to pollen from the pollinating anther. Whether pollen from the feeding anthers has reduced fertility or not was therefore not fully answered in this study. The P:O ratio was relatively low (266) for a pollinator‐dependent plant (Cruden, 1977) and would be even lower if it included only pollen from the pollinating anther. In general, P:O values are extremely variable and are not a reliable indication of pollinator dependence (Harder and Johnson, 2023). These ratios can be informative when comparing closely related species (Harder and Johnson, 2023), but this comparative approach is not yet possible in Haemodoraceae due to a lack of sufficient data for other species. Interestingly, the P:O ratio for *Dilatris ixiodes*, which places pollen on the wings of bees, is 2517 (Johnson et al., 2025), which is an order of magnitude greater than in *C. xanthorrhizos* and may indicate that the latter has a more precise system for pollen transfer.

## FUTURE DIRECTIONS

*Cubanicula* is not closely related to the two genera of Haemodoraceae in South Africa that have dimorphic enantiostyly (Hopper et al., 2009); therefore, dimorphic enantiostyly likely evolved independently in an American clade as well as in an African clade. In both clades, genera with dimorphic enantionstyly are nested among genera with monomorphic enantiostyly. It has been argued that the evolution of monomorphic enantiostyly likely preceded the evolution of dimorphic enantiostyly (Jesson and Barrett, 2003). The evolution of monomorphic enantiostyly could be due to several factors, including exploitation of lateral body parts of pollinators such as their wings or the side of the thorax for precise pollen placement and transfer, particularly if these sites are less likely to be groomed. However, the transition from monomorphic to dimorphic enantiostyly is more likely due to the advantages of cross‐pollination (through reduction of geitonogamy) than to a pollen placement opportunity. In addition to various forms of enantiomorphy, the radiation of the Haemodoraceae appears to have involved transitions in overall flower presentation. Flowers of *Cubanicula*, like those of *Wachendorfia*, have superior ovaries and are arranged on a thyrse inflorescence with nodding (pendant) side branches, resulting in flowers being presented upside‐down with the median petal lowermost, while flowers of *Dilatris* differ in having an inferior ovary and are presented in the typical monocot position with the median petal uppermost, possibly because they are both upside‐down and resupinate (Appendix S2). Understanding these complex transitions of floral architecture and development in the Haemodoraceae will require a well‐resolved phylogeny, discovery of the genes underlying the different forms of enantiomorphy, and evidence for whether or not these forms are homologous with the same genetic basis or convergent with different genetic bases. Understanding the role of selection and the function of these forms will also require additional information on the pollinators and mechanisms of pollen transfer of related groups of species, as well as studies of the implications of different types of enantiomorphy for outcrossing rates and levels of pollen discounting.

## AUTHOR CONTRIBUTIONS

S.D.J.: conceptualization, data acquisition, formal analysis, visualization, chemical analyses, photographic documentation, writing original draft, review, and editing. J.M.: data acquisition; review and editing. L.B.: data acquisition; review and editing. L.F.G.: data acquisition; review and editing. P.O.R.: review and editing. N.I.: conceptualization, data acquisition, photographic documentation; review and editing.

## Supporting information


**Appendix S1.** The sequence of bud‐flower‐closed flower‐fruit capsule maturation in *Cubanicula xanthorrhizos*.

**Appendix S2.** Floral architecture of *Wachendorfia paniculata* (A), *Dilatris ixioides* (B), and *Cubanicula xanthorrhizos* (C).

**Appendix S3.** Video of carpenter bee visiting flowers of *Cubanicula xanthorrhizos*.

**Appendix S4.** Additional interactions involving plants that share pollinators with *Cubanicula xanthorrhizos*.

**Appendix S5.** Spectrogram of audio file of carpenter bee visiting flowers of *Cubanicula xanthorrhizos*.

**Appendix S6.** The structures of compounds identified in the floral scent of *Cubanicula xanthorrhizos*.

## Data Availability

Data available at Zenodo: https://doi.org/10.5281/zenodo.16967631.
