# Supplementary material for: Dimorphic enantiostyly and its function for pollination by carpenter bees in a pollen‐rewarding Caribbean bloodwort
Source: Am J Bot. 2026 Jan 22;113(2):e70148. doi: 10.1002/ajb2.70148 (PMC12918842; doi:10.1002/ajb2.70148)

**Appendix S1.** The sequence of bud-flower-closed flower-fruit capsule maturation in *Cubanicula xanthorrhizos*.


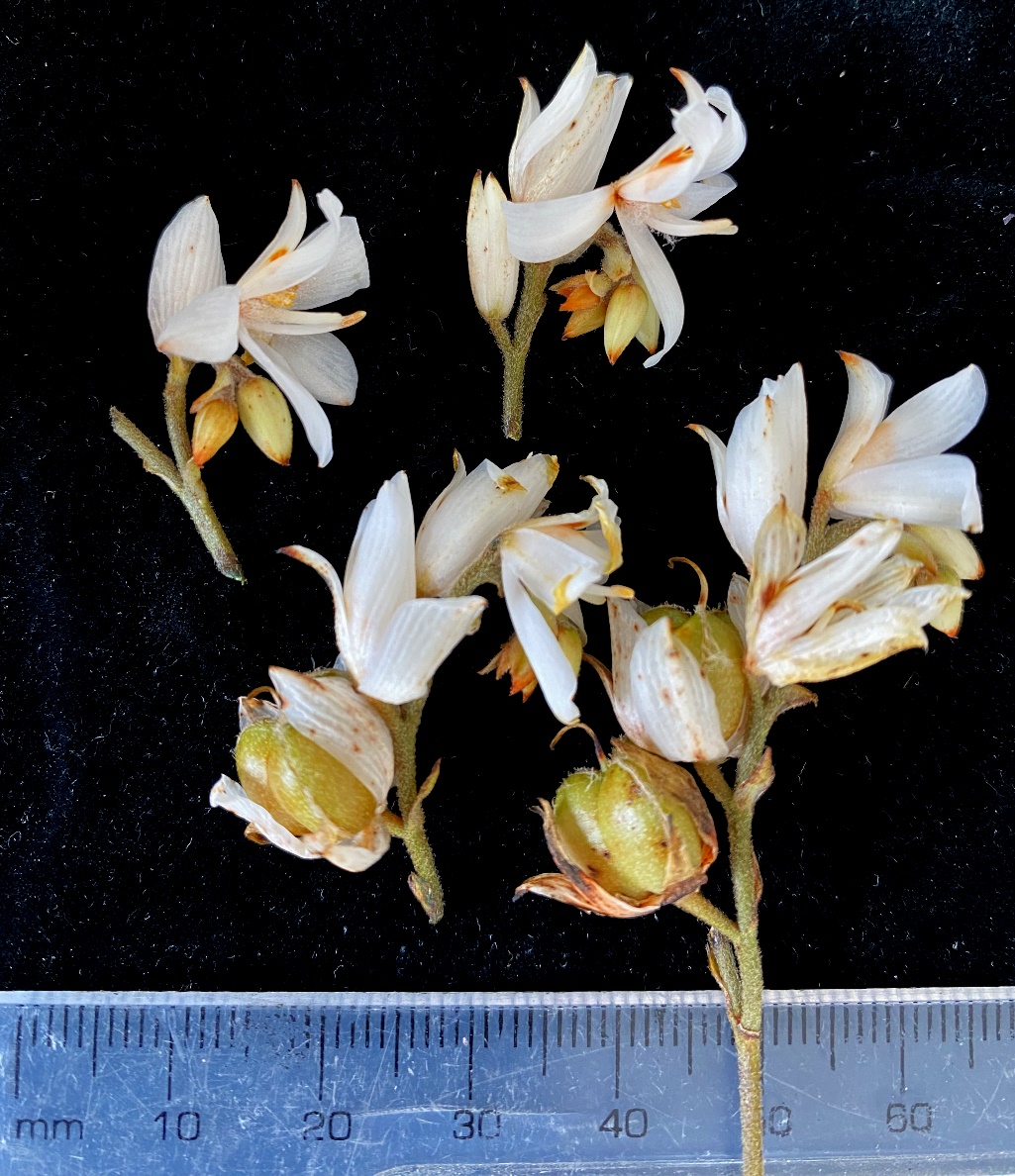

Supplement: Supplementary file 1 — Appendix S1. The sequence of bud‐flower‐closed flower‐fruit capsule maturation in Cubanicula xanthorrhizos. [file AJB2-113-e70148-s003.docx]
