# Supplementary material for: Dimorphic enantiostyly and its function for pollination by carpenter bees in a pollen‐rewarding Caribbean bloodwort
Source: Am J Bot. 2026 Jan 22;113(2):e70148. doi: 10.1002/ajb2.70148 (PMC12918842; doi:10.1002/ajb2.70148)

**Appendix S2.** Inflorescences and floral diagrams for (A) *Wachendorfia paniculata*, (B) *Dilatris ixioides*, and (C) *Cubanicula xanthorrhizos*. Abbreviations: s, sepals; p, petals. Arrowheads indicate anthers of stamens, with the same color code given for the respective stamens in the floral diagrams. The key explains symbols used for the different components of the floral diagrams. *Wachendorfia paniculata* flowers wilt after 1 day, but retain the perianth, which protects the ovary and developing fruit.


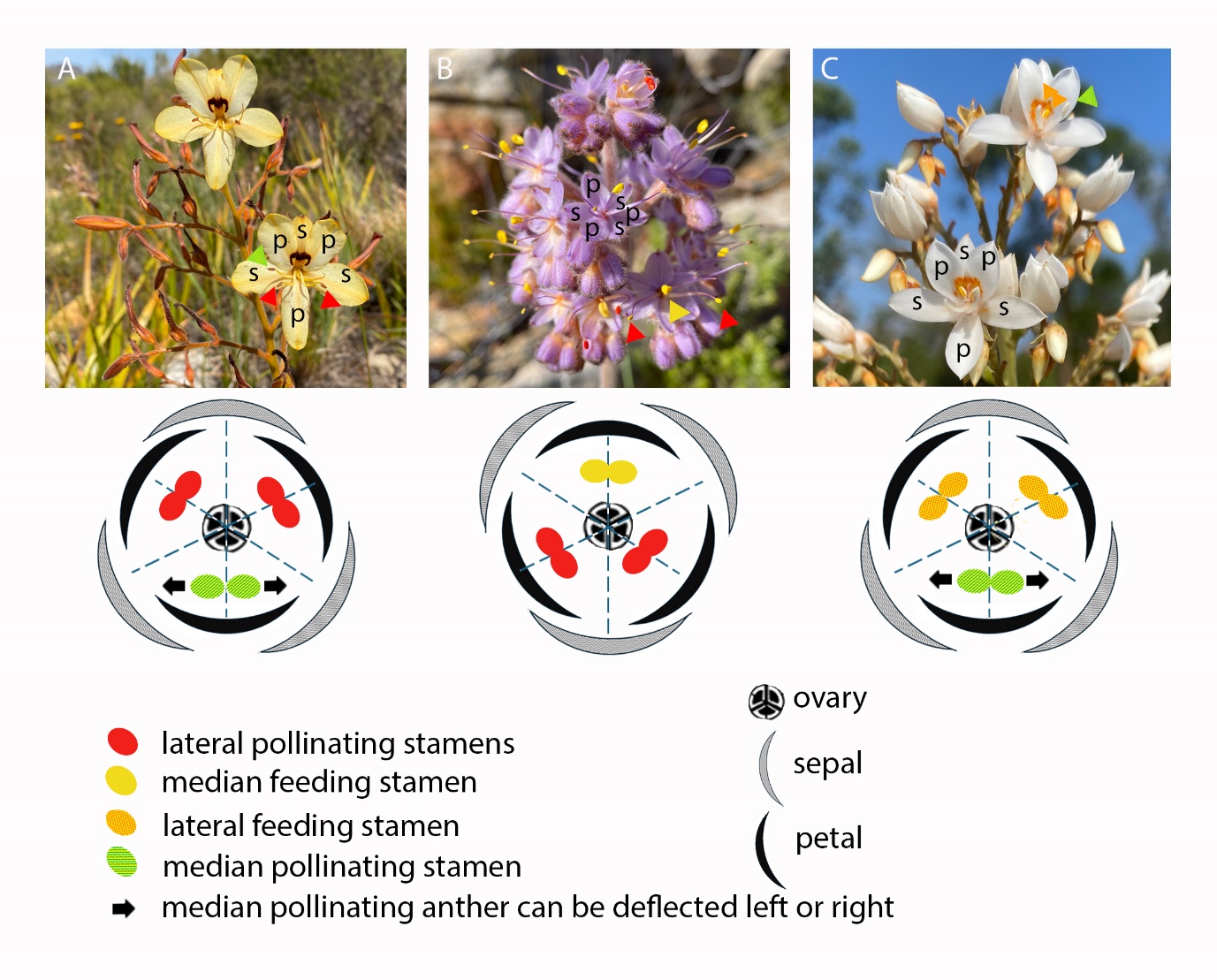

Supplement: Supplementary file 2 — Appendix S2. Floral architecture of Wachendorfia paniculata (A), Dilatris ixioides (B), and Cubanicula xanthorrhizos (C). [file AJB2-113-e70148-s001.docx]
