# Supplementary material for: Dimorphic enantiostyly and its function for pollination by carpenter bees in a pollen‐rewarding Caribbean bloodwort
Source: Am J Bot. 2026 Jan 22;113(2):e70148. doi: 10.1002/ajb2.70148 (PMC12918842; doi:10.1002/ajb2.70148)

Appendix S4. Spectrogram of audio file of carpenter bee visiting flowers of *Cubanicula xanthorrhizos.*


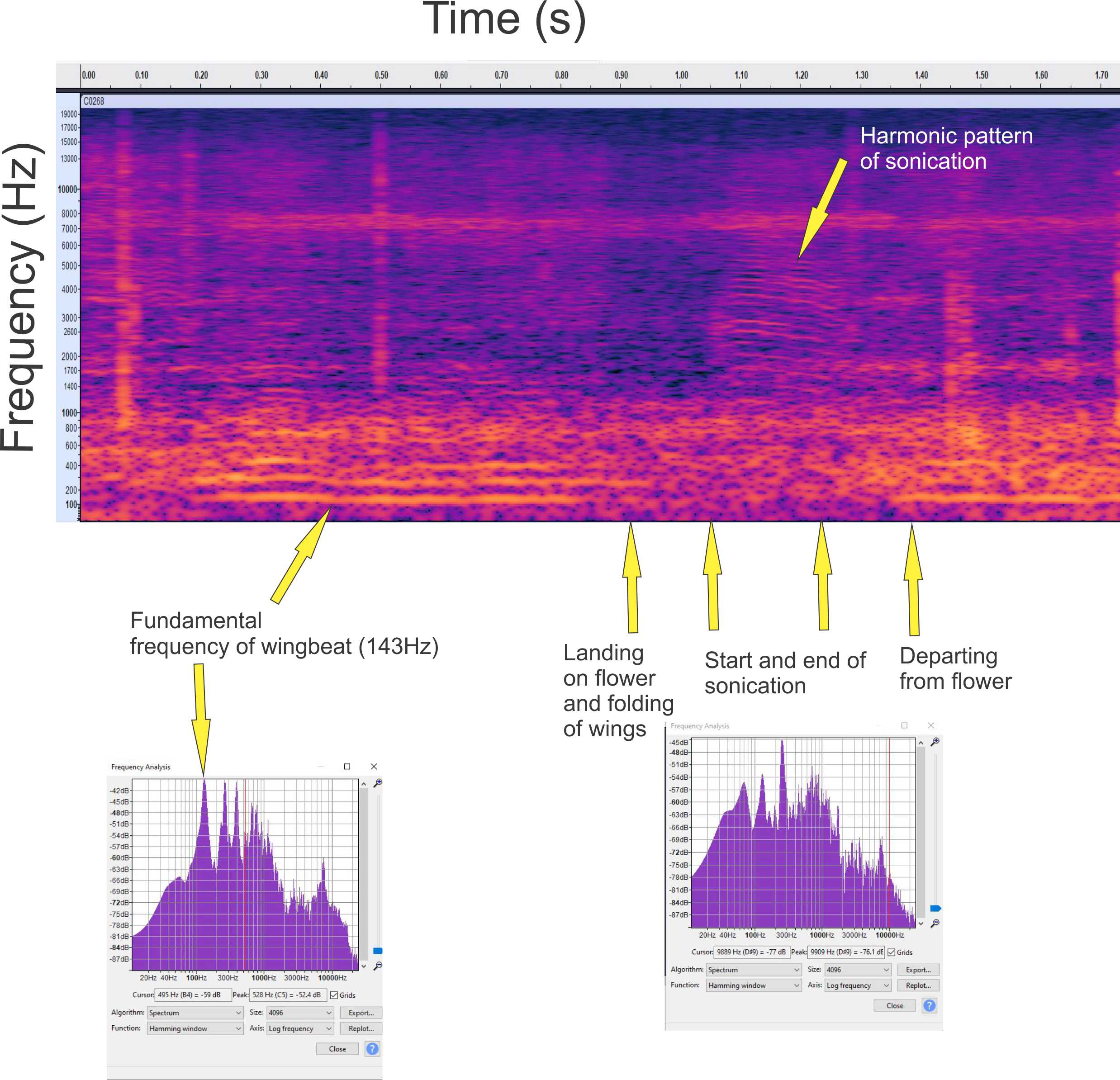

Supplement: Supplementary file 4 — Appendix S4. Additional interactions involving plants that share pollinators with Cubanicula xanthorrhizos. [file AJB2-113-e70148-s002.docx]
