# Supplementary material for: Dimorphic enantiostyly and its function for pollination by carpenter bees in a pollen‐rewarding Caribbean bloodwort
Source: Am J Bot. 2026 Jan 22;113(2):e70148. doi: 10.1002/ajb2.70148 (PMC12918842; doi:10.1002/ajb2.70148)

**Appendix S5.** Additional interactions involving plants that share pollinators with *Cubanicula xanthorrhizos*. (A) Flower of *Miconia delicatula* (Melastomataceae). (B) Oil-collecting bee *Centris poecila* buzzing-pollinating a flower of *M. delicatula*. (C) *Centris poecila* visiting a flower of *C. xanthorrhizos*. (D) *Centris poecila* collecting oil from a flower of *Byrsonima wrightiana* (Malphigiaceae). Scale bars: 5 mm.


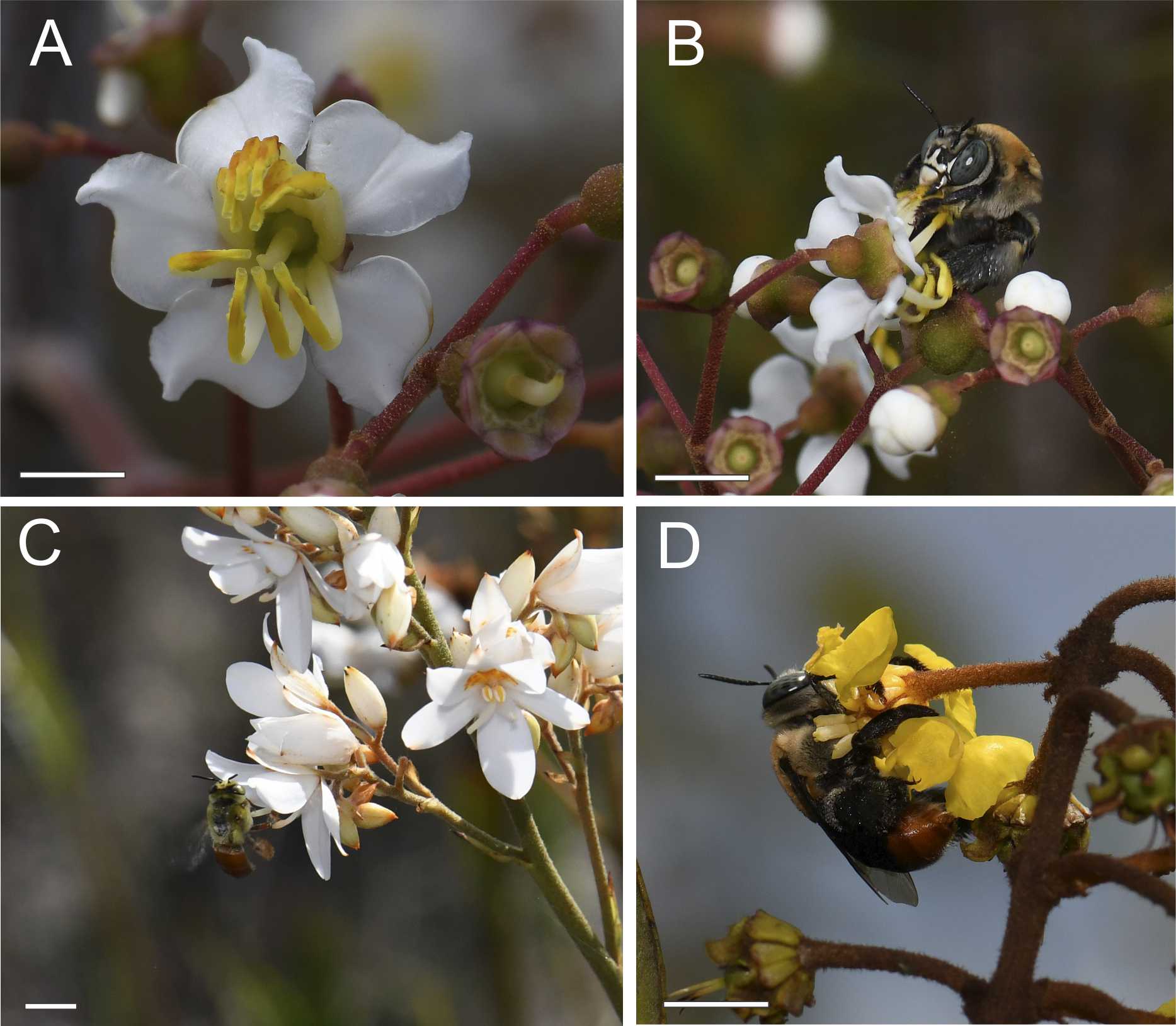

Supplement: Supplementary file 5 — Appendix S5. Spectrogram of audio file of carpenter bee visiting flowers of Cubanicula xanthorrhizos. [file AJB2-113-e70148-s006.docx]
