# Supplementary material for: Dimorphic enantiostyly and its function for pollination by carpenter bees in a pollen‐rewarding Caribbean bloodwort
Source: Am J Bot. 2026 Jan 22;113(2):e70148. doi: 10.1002/ajb2.70148 (PMC12918842; doi:10.1002/ajb2.70148)

**Appendix S6.** Structures of compounds identified in the floral scent of *Cubanicula xanthorrhizos*.


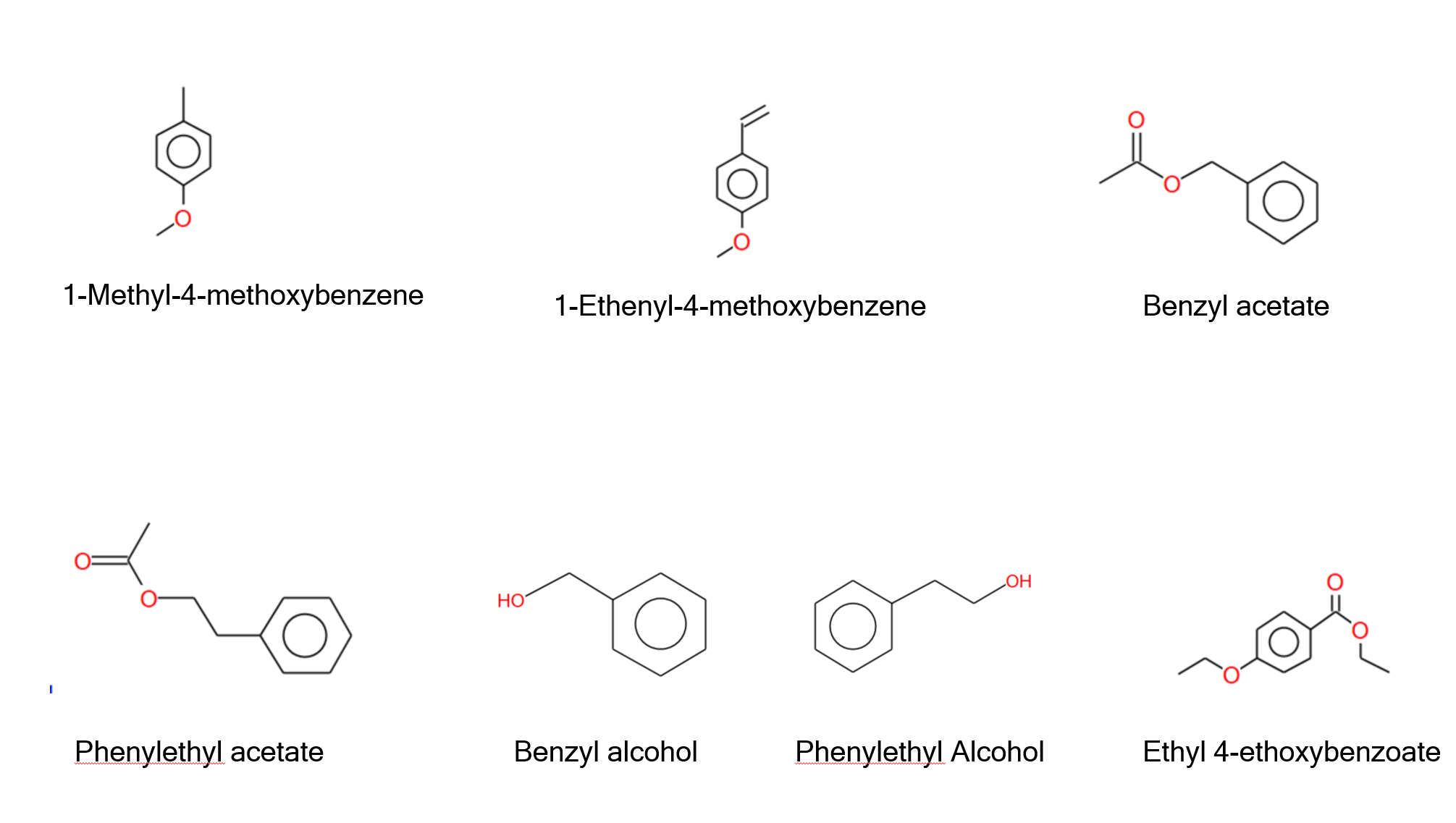

Supplement: Supplementary file 6 — Appendix S6. The structures of compounds identified in the floral scent of Cubanicula xanthorrhizos. [file AJB2-113-e70148-s004.docx]
